# Role of AMPK***α*** in Skeletal Muscle Glycometabolism Regulation and Adaptation in relation to Sepsis

**DOI:** 10.1155/2014/390760

**Published:** 2014-06-29

**Authors:** Xia Zheng, Mi Xu, Qiang Fang

**Affiliations:** Intensive Care Unit, The First Affiliated Hospital, College of Medicine, Zhejiang University, 79 Qingchun Road, Hangzhou 310003, China

## Abstract

*Background.* AMP-activated protein kinase (AMPK) and the translocation of glucose transporter 4 (GLUT4) protein always involve disturbance of carbohydrate metabolism. *Objective.* To determine whether the change of blood glucose in the early stage of septic rat is associated with the alteration of AMPK*α* protein expression and GLUT4 protein translocation expression. *Methods.* Animal models of sepsis were induced by tail vein injection of LPS in Wistar rats. The dynamic values of blood glucose within 2 hours after injection of LPS were observed. AMPK*α* protein and GLUT4 protein translocation in different tissues (such as soleus muscle and extensor digitorum longus) were assessed by western blot. *Results.* Blood glucose levels appeared to rise at 0.5 h after injection of LPS, arrived the peak value at 1 h, then fell at 1.5 h and 2 h Animals in LPS group experienced the increase of phos-AMPK*α* protein and GLUT4 protein translocation expression in soleus muscle and extensor digitorum longus. *Conclusion.* The dynamic change of blood glucose, represented in a form of initiative increase and subsequent decrease in the early stage of sepsis, may be related to glycometabolism disorder in the skeletal muscle, coming down to enhancement of GLUT4 translocation expression promoted by activation of AMPK*α*.

## 1. Introduction

Sepsis is a serious medical condition that is characterized by a whole-body inflammatory state, resulting from the systemic response to bacterial infection. If the bacteria die, the endotoxin will be released into the bloodstream. Sepsis remains one of the leading causes of morbidity and mortality in critically ill intensive care unit patients [1]. The systemic administration of lipopolysaccharide (LPS), an outer component of the gram-negative bacterial wall, has been applied as an experimental model to mimic some of the clinical findings of human septic shock [2]. The kind of vicious stimulus, at the same time, leads to severe metabolic disorder. Baseline hyperglycemia, including stress-induced hyperglycemia, is common in patients with severe sepsis. Similarly, stress-induced hyperglycemia is associated with adverse outcomes in septic patients [3–7]. In a 2001 study of critically ill intensive care unit (ICU) patients, van den Berghe and associates demonstrated that aggressive insulin therapy to maintain blood glucose between 4.4 and 6.1 mmol/L reduced mortality from 8.0% with conventional treatment to 4.6%, a relative reduction of 42% [3]. Mortality reduction in the intensive insulin treatment group was attributed to lower rates of organ failure and bacteremia. Mackenzie and colleagues recently reported that when intensive glycemic control was managed by the bedside nurse, average morning glucose concentration was 7.0 ± 2.4 mmol/L, but 42% of patients suffered hypoglycemic episodes, defined as a serum glucose <2.2 mmol/L [8]; thus studies recommend a cautious approach to the control of glucose levels in acutely ill emergency department patients, with a target glucose of below 8 to 9 mmol/L [9]. In present, we only try to control blood glucose with insulin therapy. In fact these metabolic effects induced by 5′-adenosine monophosphate-activated protein kinase (AMPK) are associated with lowering blood glucose levels in hyperglycemic individuals [10]. AMPK is widely present in eukaryotic cells, sensing the changes of cellular energy metabolism, known as the “cellular energy regulator.” Impaired glucose metabolism regulated by activated AMPK is the response to cellular stress, such as exercise, hypoxic stress, and ischemic stimulus [11–14]. It is well known that sepsis is a sophisticated morbid process and this unique model of sepsis induced by LPS always relates to the change of AMP/ATP ratio, ischemia, hypoxia, nutrition, and metabolic disorders. Therefore, as a new target for antidiabetic drugs, AMPK expression in sepsis should be noted.

Recent study showed that patients with type 2 diabetes were more prone to develop dysregulated glucose disposal, which was associated with altered AMPK phosphorylation in skeletal muscle [15]. GLUT4 is a glucose transport protein found in fat and striated muscle cells [16]. When carbohydrates are ingested, the major cellular mechanism that diminishes blood glucose is insulin-stimulated glucose transport into skeletal muscle. Skeletal muscle both stores glucose as glycogen and oxidizes it to produce energy following the transport step. The principal glucose transporter protein that mediates this uptake is GLUT4, which plays a key role in regulating whole body glucose homeostasis [17]. When insulin receptor is activated, it induces the GLUT4 protein to move from reserves held inside cells. GLUT4 can also be recruited to the cell surface through muscle contraction. In the absence of insulin or muscle contraction, GLUT4 is stored in vesicles within the cell. In addition to insulin, skeletal muscle glucose transport is possible stimulated by other media or by other pathways. AMPK is really another known regulator of glucose metabolism in skeletal muscle [18]. Activation of AMPK in muscle leads to an increase in glucose transport, accompanied by increased translocation of GLUT4 to the plasma membrane [19]. Therefore, as the important targets which always involve disturbance of carbohydrate metabolism, whether AMPK and the translocation of GLUT4 protein expression appear to change to adapt the stress hyperglycemia in early stage of sepsis still needs to be paid attention to. Thus the present study is designed to explore whether the acute blood glucose dynamic changes are partly based on translocation of GLUT4 regulated by AMPK signal pathway in the early stage of sepsis.

## 2. Materials and Methods

### 2.1. Main Materials

Anti-Phos-AMPK*α*-Thr172 antibody and anti-AMPK*α* antibody were purchased from the U.S. Cell Signaling, Inc.; anti-GLUT4 antibody was obtained from Santa Cruz Biotechnology; anti-*α*-tubulin antibody was obtained from Merck Millipore, Billerica, MA; lipopolysaccharide (LPS, Escherichia coli 0111: B4) was purchased from USA Sigma Company; insulin kit was purchased from the U.S. Adlitteram Diagnostic Laboratories Inc.; Membrane Protein Extraction Kit was purchased from the Fermentas International Inc.

### 2.2. Animal Model

12 healthy male Wistar rats (8 weeks old, 200 to 250 g) were purchased from Experiment Animal Center of Chinese Academy of Sciences in Shanghai (SCXK (Shanghai) 2007-0005). The rats were divided into two groups: LPS group (received LPS 5 mg/kg (concentration of 2 mg/mL) by tail vein injection, to establish the septic rat model) and control group (given normal saline (NS) 2.5 mL/kg by tail vein injection) [20]. Body temperature of the rat was measured using the rectal probe. The procedures in our experiments were approved by the Animal Care and Use Committee of Zhejiang University, China.

### 2.3. The Determination of Blood Glucose and Insulin Levels

Blood glucose levels were determined at 0 h, 0.5 h, 1 h, 1.5 h, and 2 h after injection of LPS or NS with an Accu-chek glucometer (Roche, Mannheim, Germany) from tail-bled samples (made with a needle stick). At 2 hours, anesthesia was executed by 3% pentobarbital sodium (0.15 mL/100 g) intraperitoneal injection. 4–6 mL blood was taken from carotid artery; serum was segregated and stored at −20°C for measurement of insulin level. Insulin levels were determined using an Ultrasensitive Insulin ELISA kit according to the manufacturer's instructions.

### 2.4. Western Blot

The samples of heart, liver, soleus muscle, and extensor digitorum longus were frozen into liquid nitrogen and stored. 100 mg of each tissue was homogenized in 1 mL modified lysis buffer (0.3 mol/L sucrose, 10 mmol/L imidazole, 10 mmol/L sodium metabisulfite, 1 mmol/L DTT, 0.3 mmol/L PMSF) [21]. The protein concentration was determined by the Bradford method.

Western blot analysis of AMPK*α* and Pho-AMPK*α* protein and *α*-tubulin were performed in heart, liver, soleus muscle, and extensor digitorum longus, while western blot analysis of GLUT4 was performed only in soleus muscle and extensor digitorum longus. Aliquots containing the protein for Phos-AMPK*α*-Thr172, AMPK*α*, GLUT4, and *α*-tubulin were loaded on the SDS-polyacrylamide gel with 10% acrylamide separating gel, respectively, and separated by electrophoresis for 30 min. The separated Phos-AMPK*α*-Thr172, AMPK*α*, GLUT4, and *α*-tubulin proteins were electrophoretically transferred onto nitrocellulose membranes (Amersham Life Science). All of the membranes were incubated at 4°C overnight with anti-Phos-AMPK*α*-Thr172 antibody (1 : 1000), or anti-AMPK*α* antibody (1 : 1000) or anti-GLUT4 (1 : 3000), or anti-*α*-tubulin antibody (1 : 1000) in 5% Carnation instant milk/TBS. After incubating with a secondary antibody (1 : 500) (Beijing Zhongshan Biotechnology, China) in 5% Carnation instant milk-TBS-Tween 20, the blots were developed using enhanced chemiluminescence according to the manual (Biological Industries, Beit Haemerk LTD, Israel) and exposed to X-ray film [22]. Normalization of protein expression was carried out using *α*-tubulin as control.

### 2.5. GLUT4 Translocation Analysis

Preparation of plasma membrane fraction from the skeletal muscles was performed as described by Dombrowski et al. [23]. Briefly, three grams of the SOL or EDL muscles were homogenized in 10 mM sodium bicarbonate, 0.25 M sucrose, 5 mM sodium azide, and 100 *μ*M PMSF. The homogenate was subjected to specific centrifugations for subcellular fractionation. The crude membrane was separated from homogenized tissue by use of triple centrifugation at 1200, 9000, and 19 000 ×g, respectively. The plasma membrane fractions were further separated by sucrose density-gradient centrifugation (25%, 32%, and 35%) at 150 000 ×g for 16 h. The plasma membrane GLUT4 (m-GLUT4) protein was collected from the fraction of 25% sucrose solution, subjected to 190 000 ×g for 60 min, and analyzed by Western blot. Immunoblotting of the tissue protein extracts was performed using anti-GLUT4 antiserum (1 : 3000). The blotted protein was quantified using quantity one software system [24–26].

### 2.6. Statistical Analysis

Data were reported as means plus or minus Standard Deviation (SD). The various kinds of indexes between control group and LPS-treated groups were compared using analysis of one-way ANOVA with SPSS 16 software. Values were considered significantly different when *P* < 0.05.

## 3. Results

### 3.1. General State of the Rats

**Figure 1:**
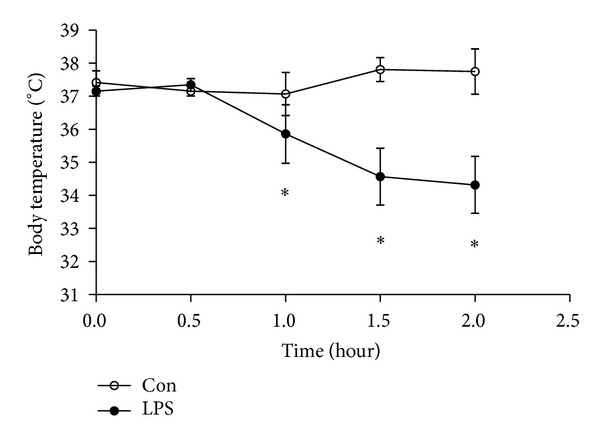
Change of body temperature induced by LPS. The change in body temperature of the rat was dynamically measured at 0 h, 0.5 h, 1 h, 1.5 h, and 2 h after injection of LPS or NS. Data are expressed as mean ± S.D. (*n* = 6 per group). *P < 0.05, **P < 0.01 LPS group (LPS) versus control group (Con).

Rats in control group were still active as usual, with good state, while those in LPS group showed mental weaknesses, physical inactivity dull coat, breathing frequently, greedy overdrink, and abnormal body temperature. Body temperature represented in a form with a rapid decline after 0.5 h and then kept lower within 2 h. In an hour after treatment, there was statistically significant effect on half-hourly body temperature between LPS group and control group (35.86 ± 0.88 versus 37.07 ± 0.65 at 1 h, *P* < 0.05; 34.57 ± 0.86 versus 37.81 ± 0.36 at 1.5 h, *P* < 0.05; 34.32 ± 0.86 versus 37.75 ± 0.69 at 2 h, *P* < 0.05, separately) (see Figure 1).

### 3.2. Dynamic Change of Blood Glucose

**Figure 2:**
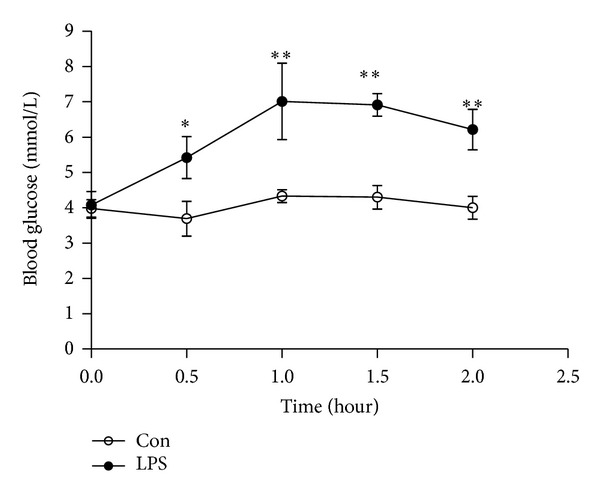
Change of blood glucose induced by LPS. Blood glucose levels were determined at 0 h, 0.5 h, 1 h, 1.5 h, and 2 h after injection of LPS or NS. Data are expressed as mean ± S.D. (*n* = 6 per group). *P < 0.05, **P < 0.01 LPS group (LPS) versus control group (Con).

Blood glucose levels appeared to rise at 0.5 h after injection of LPS, arrived the peak value at 1 h, then fell at 1.5 h and 2 h in LPS group. In half an hour after treatment, there was statistically significant effect on half-hourly blood glucose between LPS group and control group (3.69 ± 1.21 versus 5.42 ± 1.45 at 0.5 h, *P* < 0.05; 4.33 ± 0.45 versus 7.01 ± 2.65 at 1 h, *P* < 0.01; 4.30 ± 0.82 versus 6.91 ± 0.79 at 1.5 h, *P* < 0.01; 4.00 ± 0.79 versus 6.21 ± 1.40 at 2 h, *P* < 0.01, separately) (see Figure 2).

### 3.3. Changes of Plasma Insulin

**Figure 3:**
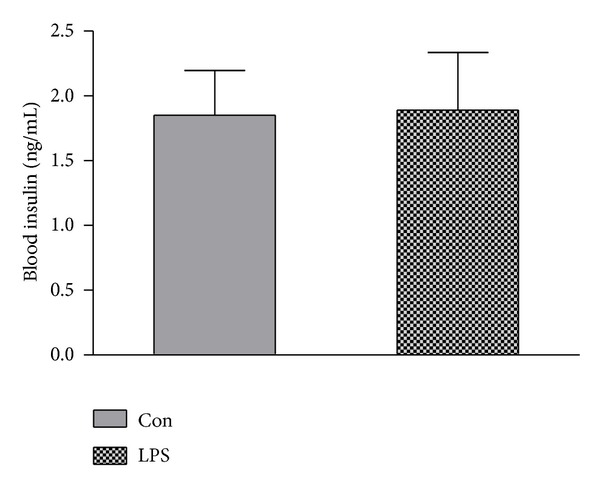
Change of blood insulin induced by LPS. At 2 hours after injection of LPS or NS, 4–6 mL blood was taken from carotid artery; serum was segregated for measurement of insulin level. Data are expressed as mean ± S.D. (*n* = 6 per group). *P < 0.05, **P < 0.01 LPS group (LPS) versus control group (Con).

At 2 hour, there was no significant different of serum insulin level between LPS and control group (1.85 ± 0.85 versus 1.89 ± 1.09, *P* > 0.05) (see Figure 3).

### 3.4. Effects of LPS on Protein Expression of Phos-AMPK*α* and AMPK*α*

**Figure 4:**
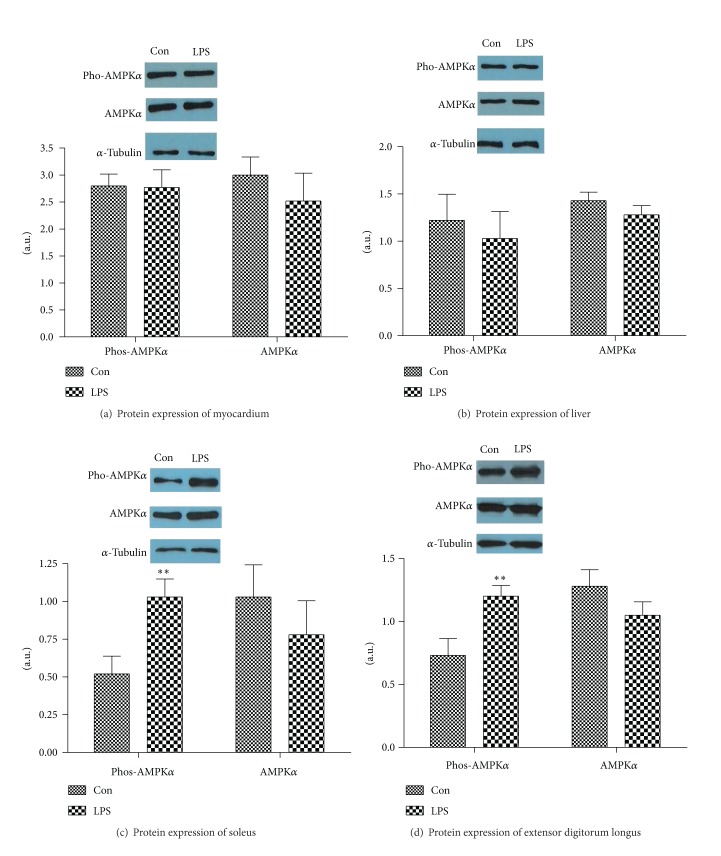
The effects of LPS on the protein expression of phos-AMPK*α* and AMPK*α* in different tissues: heart (a), liver (b), soleus muscle (c), and extensor digitorum longus (d). Equal amounts of protein were subjected to electrophoresis and immunoblotted, as described. Data were represented as mean ± S.D. (*n* = 6, per group) *P < 0.05,  **P < 0.01 LPS group (LPS) versus control group (Con).

LPS failed to alter the protein expression of AMPK*α* in different tissues (soleus muscle 0.78 ± 0.55 versus 1.03 ± 0.52, *P* > 0.05; extensor digitorum longus 1.05 ± 0.26 versus 1.28 ± 0.32, *P* > 0.05; liver 1.28 ± 0.24 versus 1.43 ± 0.22, *P* > 0.05; and myocardium 2.52 ± 1.26 versus 3.00 ± 0.82, *P* > 0.05). No impact of LPS on abundance of Phos-AMPK*α* proteins of cardiac (2.77 ± 0.80 versus 2.80 ± 0.53, *P* > 0.05) and liver (1.03 ± 0.70 versus 1.22 ± 0.68, *P* > 0.05) was exhibited in this study. However, LPS induced significant increase of Phos-AMPK*α* proteins in soleus muscle (1.03 ± 0.29 versus 0.52 ± 0.29, *P* < 0.01) and extensor digitorum longus (1.20 ± 0.21 versus 0.73 ± 0.33, *P* < 0.01) (see Figure 4)

### 3.5. Effects of LPS on Expression of GLUT4 Protein Translocation in Skeletal Muscle

**Figure 5:**
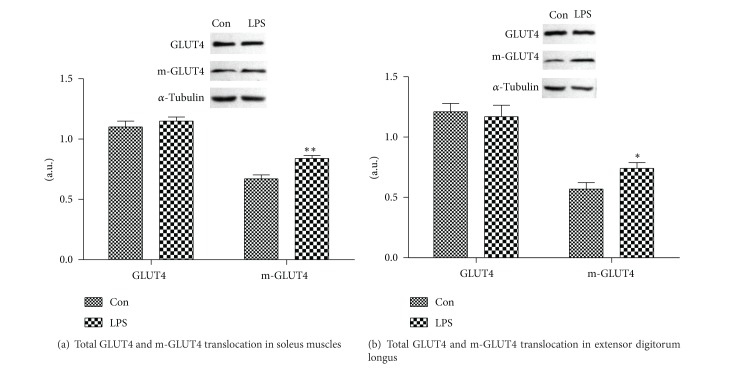
The effect of LPS on total GLUT4 and m-GLUT4 translocation in skeletal muscle (soleus muscle or extensor digitorum longus). Preparation of plasma membrane fraction from the skeletal muscles was performed. The proteins were analyzed by western blot. Results were normalized by *α*-tubulin, and the m-GLUT4 was normalized by the total protein. Data were represented as mean ± S.D. (*n* = 6, per group) *P < 0.05,  **P < 0.01 LPS group (LPS) versus control group (Con).

GLUT4 and m-GLUT4 expression levels in skeletal muscle by Western blot. As shown in Figures 5(a) and 5(b), no significant differences in total GLUT4 protein in soleus muscle (1.15 ± 0.08 versus 1.10 ± 0.12, *P* > 0.05) and extensor digitorum longus (1.17 ± 0.23 versus 1.21 ± 0.17, *P* > 0.05) were observed between LPS and control group. However, LPS induced the increase in the expression of GLUT4 protein translocation of soleus muscle (0.84 ± 0.06 versus 0.67 ± 0.08, *P* < 0.01) and extensor digitorum longus (0.74 ± 0.12 versus 0.57 ± 0.13, *P* < 0.05).

## 4. Discussion

Sepsis is a kind of severe illness, caused by infection in the body. When inflammation overwhelms the host, simple infections will develop into sepsis. Sepsis is associated with various metabolic and endocrine disorders that can be confusing [27]. On the one hand, metabolic disorders in sepsis express high catabolic state with increased energy consumption. These patients often exhibit a well-defined endocrine and metabolic adaptive response to stressor agents, partly characterized by incremented resting energy expenditure (hypermetabolism, which is believed to signify increased energy requirements) [28]. That is to say, a cardinal manifestation is hyperglycemia [27]. On the other hand, some metabolic pathways were demolished in sepsis. For example, prolonged sepsis and exposure to an inflammatory milieu decreases muscle protein synthesis and reduces muscle mass [29]. Hyperglycemia is frequently easily observed during bacterial infection and it is a marker of a poor clinical outcome in critically ill patients. Lipopolysaccharides (LPS) of the cell wall of Gram (−) bacteria trigger inflammation, which is associated with marked changes in glucose metabolism; thus recently more and more attention has been paid to LPS-induced glucose metabolism disorder, which is a prominent pathological problem [30, 31].

Our experiment showed that, blood glucose levels were elevated in 0.5 h after injection of LPS, and there was statistically significant effect on half-hourly blood glucose between LPS group and control group from 0.5 h to 2 h. In fact, physical trauma, surgical-site infection, and many forms of severe stress can temporarily increase glucose levels [32–34]. Even only hypothermia can have the “perverse result.” For example, adverse events may develop when a patient is treated with hypothermia [35]. One of the adverse events associated with hypothermic therapy is a decrease in insulin sensitivity and insulin secretion, which can lead to hyperglycemia [35]. In our experiment, body temperature represented in a form with rapidly decline after 0.5 h induced by LPS, then kept lower within 2 hours. In fact, sepsis is a complex pathological process, and multiple factors are involved in abnormally high blood sugar. So far the mechanism of stress hyperglycemia in early stage of sepsis still leaves a puzzle. Stress in early stage of sepsis can increase sympathetic nerve activity, then the autonomic nervous system regulated adrenocortical function, and catecholamines facilitated the action of glucocorticoids. In addition to pituitary adrenocorticotropin, there are other extrapituitary factors regulating adrenal steroidogenesis in septic shock [36]. Similarly, glucagon was triggered to increase the level of glucose [36, 37]. In present, even plasma glucose >120 mg/dL in the absence of diabetes is a clinical sign of sepsis. Of course, hyperglycemia may be associated with increased mortality, while strict regulation of glucose levels has been found to decrease mortality and length of stay in the ICU. Now we only try to control blood glucose with insulin therapy. However, in our experiment, no significant changes in insulin levels were observed after 2 h of LPS injection, similar with the results of D. T. Yates et al. [38]. It is speculated that only 2 hours after LPS injection were too little time to finish the desired changes of insulin levels. However, in 2 hours after LPS injection, blood glucose levels significantly fell a long way from their peak simultaneously. Thus, rather than insulin action, another way, such as 5′-adenosine monophosphate-activated protein kinase (AMPK), maybe become the pathway to affect the self-regulation of blood glucose after IV bolus of LPS.

It is well known that as a highly conserved serine/threonine protein kinase, AMPK can become the important metabolic stress protein kinases, constituted by *α*, *β*, and *γ* 3 subunit. Once activated, AMPK phosphorylates several downstream substrates, the overall effect of which is to switch off ATP-consuming pathways (e.g., fatty acid synthesis and cholesterol synthesis) and to switch on ATP-generating pathways (e.g., fatty acid oxidation and glycolysis) [39]; thus the phosphorylation of AMPK become a central link of cellular energy regulation. And AMPK on the regulation of carbohydrate metabolism is mainly reflected the promotion of glucose uptake, glycolysis, inhibit gluconeogenesis, and glycogen synthesis. Even in recent years AMPK has become an attractive pharmacological target for the treatment of insulin resistance and type 2 diabetes-associated dyslipidaemia [40].

Patients with sepsis have a hypermetabolic and hypercatabolic state, which can be represented by increased oxygen demand on the body tissue and reduced oxygen consumption because of microcirculatory disturbance, thus ATP generation is decreased, the AMP to ATP ratio is increased, and AMPK is activated at last. It is well known that AMPK activation favors carbohydrate metabolism under some certain conditions. For example, activation of AMPK is thought to mediate, at least partially, the increases in skeletal muscle fatty acid oxidation and glucose transport that occur during acute exercise [41]. AMPK activation is a complex and elaborates the regulating process. As the action sites, *α*-subunit 172 threonine can provide the key role on AMPK activation. Therefore, activation of AMPK requires its phosphorylation at Thr 172 site [42]. Our experiment showed that AMPK*α* and Phos-AMPK*α* in myocardium and liver tissue of septic rats had no significant difference, compared with those in control group, after 2 h of LPS injection. However, the levels of Phos-AMPK*α* in the soleus muscle and extensor digitorum longus were significantly increased, although the expression of AMPK*α* was not impaired. In association with the alteration of blood glucose, it was speculated AMPK activation in exercising muscles could take part in the glycometabolism process in early stage of sepsis, while the metabolic capacity of blood glucose was not relate to AMPK activation in myocardial and liver tissue.

The signaling mechanism, downstream of AMPK, which regulates muscle glucose transport, is unclear in septic rat. Previous studies showed that, in skeletal muscle, AMPK was activated by exercise/contraction, metformin, and thiazolidinediones resulting in an increase in glucose uptake [43]. The skeletal muscle is the main peripheral tissue of glucose metabolism. The rate-limiting step of glucose metabolism is the pathway of glucose into skeletal muscle cells, which requires direct involvement of GLUT4 on the cell membrane. In cell culture, Edward O. Ojuka et al. [44] found AICAR (5-amino-4-ammonia ribonucleotide formyl imidazole), as AMPK activator, could activate AMPK to divert GLUT4 within the cell toward cytomembrane. And Bergeron et al. [45] showed that, in the quiet state, AICAR could activate AMPK, promoting GLUT4 protein translocation in cell membrane, which would increase glucose transport and uptake in skeletal muscle.

The adjustment mechanism of AMPK has been confirmed in state of exercise. On the one hand, islet *β*-cell insulin receptor, insulin-like growth factor receptor and peripheral insulin receptors mRNA expression, and protein expression can be adjusted by activation of AMPK [46]. On the other hand, AMPK can be activated by noninsulin signals in skeletal cells, so that GLUT4 within cytoplasm will shift to Cytolemma and various plasma membrane, enhancing the capacity of glucose transport [47]. In the experiment, LPS induced the increase in the expression of GLUT4 protein translocation of soleus muscle and extensor digitorum longus. Prompt decline in blood glucose at this time may be related to activation of AMPK regulation of skeletal muscle glucose metabolism [44, 48]. Because the result in this study showed that the level of insulin in LPS group did not alter; thus, in the early stage of sepsis, GLUT4 protein translocation by noninsulin dependent pathway can be actually a mechanism for glucose metabolism in skeletal muscle.

Generally skeletal muscle fibers are a mixture of 3 types of muscle fibers: type I (red fibers, slow-twitch, and slow oxidative), type II a (red fibers, fast-twitch, and fast oxidative), and type II b (white fibers, fast-twitch, fast glycolytic). Soleus muscle fibers mainly belong to type I, while extensor digitorum longus muscle fiber belongs to type II. To the different muscle fiber types, AMPK response is various. AMPK may be involved in the signal transduction pathway induced by fast muscle movement, while AMPK is not related to the slow-twitch fibers [49–51]. But in this experiment, Phos-AMPK*α* expression and GLUT4 protein translocation expression of the soleus muscle and extensor digitorum longus all increased in 2 h after LPS injection. Therefore, it is deduced that, in early stage of acute sepsis, the effect of AMPK on glucose metabolism in skeletal muscle may not be related to muscle fiber type.

In conclusion, the dynamic changes of blood glucose appeared to be an increase at first and then a drop in early stage of acute sepsis. The changes of blood glucose have no bearing on glucose metabolism in cardiac muscle and liver tissue. Non-insulin-dependent AMPK signaling pathway can increase the expression of GLUT4 protein translocation to promote skeletal muscle glucose metabolism. Activation of AMPK on the regulation of glucose metabolism in skeletal muscle has no relation to muscle fiber type.
